# Knowledge, attitudes and practices regarding tuberculosis care among health workers in Southern Mozambique

**DOI:** 10.1186/s12890-016-0344-8

**Published:** 2017-01-05

**Authors:** Andrés Noé, Rafaela M. Ribeiro, Rui Anselmo, Maria Maixenchs, Layce Sitole, Khatia Munguambe, Silvia Blanco, Peter le Souef, Alberto L. García-Basteiro

**Affiliations:** 1 School of Paediatrics and Child Health, University of Western Australia, Crawley, Australia; 2 Centro de Investigação em Saúde de Manhiça (CISM), CP 1929 Maputo, Mozambique; 3 Maternal and Infant Health Service, Manhiça District, Maputo, Mozambique; 4 Faculdade de Medicina, Eduardo Mondlane University (UEM), Maputo, Mozambique; 5 ISGlobal, Barcelona Ctr. Int. Health Res. (CRESIB), Hospital Clínic - Universitat de Barcelona, Barcelona, Spain; 6 Amsterdam Institute for Global Health and Development (AIGHD), Amsterdam, The Netherlands

**Keywords:** Tuberculosis, Control, Health care workers, Mozambique, Knowledge, Attitudes, Practises

## Abstract

**Background:**

Tuberculosis (TB) control is more likely to be achieved if the level of knowledge regarding TB is increased among health workers managing high-risk groups. No formal assessments regarding knowledge, attitudes and practises of health workers about TB have been published for Mozambique, a country facing challenges in the fight against TB, with a fragile health system and considerable work overload of health personnel. The main objective of the study was to determine the level of knowledge, identify attitudes and assess practices regarding TB care and control among health care workers of the district of Manhiça.

**Methods:**

A descriptive cross-sectional study was performed through the use of a specifically designed Knowledge, Attitudes and Practices (KAP) questionnaire in the district of Manhiça, a high tuberculosis and HIV burden rural area in Southern Mozambique. In this district, 14 health care facilities service a population of approximately 160,000 people. The questionnaire took 30–45 min to administer with external assistance not permitted. The survey contained 79 questions pertaining to four different areas: demographics, TB knowledge, attitudes and practices.

**Results:**

The study sample included 170 health care workers. The average knowledge score was 14.89 points (SD = 3.61) out of a total possible 26 points. Less than 30% of respondents had heard of Xpert MTB/RIF®. Seventy per cent agreed there was stigma associated with TB and 48.2% believed this stigma was greater than that associated with HIV. The average practice score was 3.2 out of 9 points (35.6%, SD = 2.4).

**Conclusion:**

Health care worker’s knowledge gaps identified in this study may result in substandard patient care. Specific deficiencies in understanding existed in terms of paediatric TB and Xpert MTB/RIF® testing. The present study provides impetus for tailored TB education among health care workers from a high TB burden rural area in Southern Mozambique.

**Electronic supplementary material:**

The online version of this article (doi:10.1186/s12890-016-0344-8) contains supplementary material, which is available to authorized users.

## Background

The World Health Organisation (WHO) has deemed tuberculosis (TB) a global public health emergency since 1993 [1]. TB ranks as the leading cause of death from infectious disease [2]. In 2014, there were 9.6 million new cases and 1.5 million deaths due to TB [2]. Mozambique has been among the 22 high TB-burden countries for many years, ranking sixth in TB incidence [2]. The estimated incidence for the country is 551 per 100,000 [2].

TB control is more likely to be achieved if the level of knowledge regarding TB is increased among health care workers (HCWs) managing high-risk groups [3]. HCWs need to be adequately educated and trained in order to effectively treat TB [4]. In particular, front-line staff members are intrinsically linked to the success of TB control programs through their involvement in treatment services and supervision, and patient support (element three of WHO’s Directly Observed Treatment Short-course approach) [5]. As such, assessing the knowledge, attitudes and practices (KAP) about TB in front-line TB HCWs is essential in order to optimise difficulties, limitations and capacities for enhancement.

KAP-style surveys can be used to establish a baseline or measure intervention-related changes in a person’s understanding, related thoughts and skills. Some studies have found that TB knowledge among HCWs was in general poor, especially in terms of diagnosis and treatment [6–8]. Good understanding of TB disease management is often inadequate even in the context of recent TB training [4]. By contrast, other studies suggest that TB knowledge pre-training is satisfactory and may even improve following brief education [9–11]. While the results of a once-off training program can be inconsistent, there are examples of how periodic training and supervision can improve HCWs’ TB knowledge and skills [12, 13].

No KAP-style TB assessments among HCWs have been published in Mozambique, a country facing challenges in the fight against TB, with a fragile health system and considerable work overload of health personnel [14, 15]. Therefore we aimed to determine the level of knowledge, attitudes and practices regarding TB care among HCWs in a rural area in Southern Mozambique.

## Methods

### Study setting

We conducted a descriptive cross-sectional study over January-March 2015.

The study was conducted in the district of Manhiça, a high TB- and HIV-burden, low-resource, rural area in Southern Mozambique. [16–18] Manhiça district has very high TB incidence and TB associated mortality rates with a low case detection rate [19, 20]. In the district there is one district hospital (Hospital Distrital da Manhiça), one rural hospital (Hospital Rural de Xinavane) and 12 peripheral primary Health Care Centres (HCC). Altogether, these centres service, at the time of the survey, a population of approximately 160,000 people [21–23].

### Survey and data collection

The survey was designed selecting relevant questions from previous published studies [4, 6, 9–11], and based on current Mozambican National Tuberculosis Program (NTP) guidelines, after consensus from a team including clinicians, a social scientist and an epidemiologist with experience in TB and KAP studies. The survey was conducted in an interview format as a standard, but self-administered if the HCW repeatedly (more than two times) reported they had insufficient time for an interview. The questionnaire took 30–45 min to administer with external assistance not permitted. The survey contained 79 questions pertaining to four different areas: demographics, TB knowledge, attitudes and practices. The questionnaire was written first in English and then translated to Portuguese. The demographics collected included age, gender, profession and TB-specific training.

The knowledge section contained 31 questions and was sub-divided into three sections: transmission (8 questions), diagnosis (9 questions) and treatment of TB (14 questions). Responses were established as correct or incorrect. If participants indicated more than one choice for a question or if a question was left unanswered, the question was marked as incorrect. The attitudes section posed 29 questions encompassing the subjects of quality of education of NTP HCWs, community awareness, access and barriers to the NTP, resources devoted to the NTP, treatment adherence and local TB control program priorities. The responses to these questions were obtained using a 5-point Likert scale and were collapsed into agreement, neutral or disagreement for analysis. Practices were not directly observed, rather three common practice scenarios were presented and one open question and two multiple-choice questions were posed regarding the management of these scenarios. Additional file 1 includes the questionnaire (with possible answers) in its entirety.

### Data analysis

Completed questionnaires were double entered into an electronic database using the REDCap software (version 5.7.3 Copyright © RedCap Creative Group, Amarillo, TX, USA). Data was exported from completed questionnaires and prepared for analysis. Answers were scored and means were treated as continuous variables. Means and/or proportions with corresponding 95% confidence intervals were calculated for each question. These were then stratified by category of health care worker. The knowledge and practice scores were analysed using a one way analysis of variance test to study mean score differences among different groups. Tukey HSD test was used for pairwise comparisons where the analysis of variance (ANOVA) demonstrated a significant difference between the groups.

## Results

In total, 170 HCWs participated in the study. This represented over 90% of the HCWs in the Manhiça district. There were eight HCWs who refused to take part in the survey. The most common reason for refusal was a lack of time to participate in the study. Fifty-one per cent of respondents were female with medical technicians (medical professionals who attended technical college) and nurses being the most common profession taking part (34.1% and 30.5%, respectively). Over half of the sample worked in Manhiça District Hospital. Seventy-five per cent of respondents reported never having had TB-specific training. Table 1 illustrates the demographic characteristics of the study participants.

**Table 1:** Characteristics of health care workers included in the study

	Category	Number	Per cent
Gender	Male	38	48.7
	Female	40	51.3
	No response	92	
Time spent working	Less than one year	24	14.2
	One to five years	64	37.9
	Five to ten years	35	20.7
	More than ten years	46	27.2
	No response	1	
Profession	Medical Agent	23	14.0
	Counsellor	1	0.6
	Nurse	50	30.5
	Midwife	21	12.8
	Dentist	1	0.6
	Doctor	10	6.1
	Microscopist	2	1.2
	Medical Technician	56	34.1
	No response	6	
Level of education	Primary school	4	2.4
	Secondary school	28	17.1
	Technical college	108	65.9
	University	24	14.6
	No response	6	
Age	20 - 25	30	19.2
	26 - 30	47	30.1
	31 - 35	34	21.8
	36 - 40	12	7.7
	41 - 45	12	7.7
	46 - 50	6	3.8
	51 - 55	14	9.0
	56 - 60	1	0.6
	No response	14	
Place of work	Manhiça	90	54.2
	Xinavane	37	22.3
	Peripheral HCC	39	23.5
	No response	4	
Participated in TB training in the past?	Yes	43	25.9
	No	123	74.1
	No response	4	
TB training in past 6 months?	Yes	3	1.76
	No	166	97.6
	No response	1	
Diagnosed with active TB in the past?	Yes	14	8.4
	No	153	91.6
	No response	3	
Directly involved in TB control?	Yes	52	32.1
	No	110	67.9
	No response	8	

### Knowledge

**Fig. 1:**
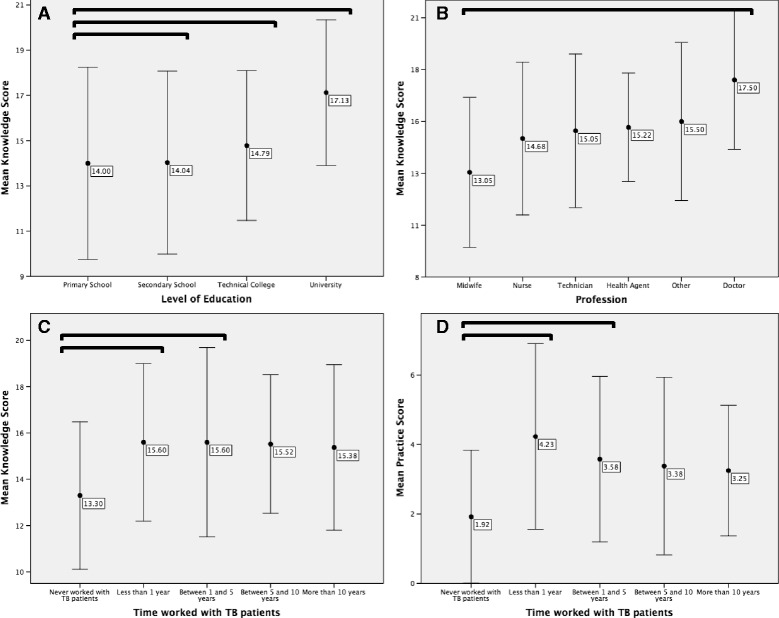
Panels **a**) Mean of knowledge score against highest level of educational attainment. **b**) Mean of knowledge score against profession. **c**) Mean of knowledge score against time worked with TB patients. **d**) Mean of practice score against time worked with TB patients. Error bars: ± 1 x ﻿SD. The square brackets denote a statistically significant difference between the two groups bounded by the bracket

The average knowledge score was 14.89 points (57.3%, standard deviation (SD) = 3.61) out of a total possible score of 26 points. Greater educational attainment levels were associated with higher knowledge scores (Fig. 1a). Respondents that had attended secondary school and technical college had lower mean knowledge scores than university-educated respondents (by 3.1 points, *p* = 0.01 and 2.3 points, *p* = 0.02, respectively).

Profession affected knowledge scores (Fig. 1b). Medical doctors had the greatest knowledge score (17.5 points, SD = 3.34) while midwives had the lowest knowledge scores (13.05 points, SD = 3.61), with this difference being statistically significant (*p* = 0.02).

Working with TB patients for one year and between one and five years were both associated with a 2.3 point increase in knowledge score (Fig. 1c), compared to having never worked with TB patients (*p* = 0.04 and *p* = 0.01, respectively). Having cared for more than 40 TB patients over the HCWs’ career was associated with a 1.57 point score increase over those who had cared for less than 10 patients (*p* = 0.049). Gender, place of work and age did not significantly affect knowledge scores.

Less than 30% of respondents had heard of Xpert MTB/RIF®. Of the 50 that were aware of this diagnostic test, 35 identified that it was a test used to diagnose TB and 7 HCWs acknowledged that it could also detect rifampicin resistance. Less than half of respondents accurately stated that paediatric TB was more difficult to diagnose than adult TB. When asked specifically, less than 25% of respondents correctly identified the difference in treatment time required between children and adults.

### Attitudes

Ninety-five per cent of respondents agreed with the statement that community engagement was essential for the control of TB. More than 90% concurred with the idea that one should know one’s own HIV serostatus. Infection control was seen as an important means to prevent the contraction of TB by 89.4% of the participants.

Seventy per cent of respondents agreed that there was a stigma associated with TB and 48.2% of them believed that this stigma was greater than that which is associated with HIV. Thirty-five per cent of the sample disagreed with the assertion, “The way I interact with TB patients contributes to TB-related stigma.” Forty-four per cent of HCWs agreed with the statement, “In Mozambique, there are many barriers to TB treatment”, with 35.9% disagreeing.

The statement “traditional or alternative medicine assists in the wellbeing of TB patients” led to a 71.8% disagreement rate.

### Practices

Overall practice competency was low with the average practice score being 3.2 points out of 9 (35.6%, SD = 2.44).

The highest level of educational attainment and profession were associated with higher mean practice scores. University educated respondents had greater practice scores (average 4.71 points, SD = 2.49) when compared to technical college educated respondents (average 2.97 points, SD = 2.37, *p* = 0.009). Doctors on average scored 2.77 and 3.50 points greater than midwives and other HCWs, respectively (*p* = 0.033 and *p* = 0.015). Those that had never worked with TB patients had lower practice scores than those that had been working with the patients for less than one year and between one and five years (2.31 points, *p* < 0.001 and 1.66 points, *p* = 0.004, respectively; Fig. 1d).

Only 26.4% correctly identified TB as one of the most likely diagnoses in a patient vignette with a persistent cough, night sweats and fatigue in the context of a close relative with similar symptoms. While 30% of professionals noted that fixed dose combinations was used in the intensive phase of TB treatment, only 50% of these could correctly name all four drugs used, as well as the amount of time that this phase lasts. Over 58% of the participants could not identify the drugs or the time frame for the maintenance phase of treatment. Only 20 respondents correctly identified the course of action to take when a patient presents with jaundice after recently commencing TB intensive phase treatment. While the majority of respondents (79 HCWs) noted that they would monitor the liver enzymes, they failed to relate the occurrence of jaundice to TB treatment.

## Discussion

This study, identifies key areas for training on TB and stresses the need for continuous re-training of all TB front-line health personnel. The present study’s main findings were: a) knowledge regarding TB patient characteristics, diagnosis and treatment was poor among HCWs; b) specific deficiencies in understanding existed in terms of paediatric TB and Xpert MTB/RIF® testing; c) there was disagreement in attitudes regarding stigma and traditional/alternative medicine, and d) practice competencies were poor.

Across all professions, knowledge in the study sample relating to TB was low. While this finding contrasts some studies from larger African centres [24–26], it is consistent with similar rural low-resource high-TB burden samples in Uganda, Tanzania, South Africa and Kenya [7, 8, 27, 28]. Although context dependent, the low knowledge might reflect the difficulty of training HCWs and keeping them updated with current NTP guidelines in low resource settings [29]. As would be expected, doctors had the highest knowledge scores, and nurses and midwives were the two poorest performing professions in the knowledge section. Naidoo et al. also noted the knowledge gap between professions [30]. This has important clinical implications; in Manhiça doctors develop care plans that nurses then implement, as a result patient care may be adversely affected if a nurse fails to comprehend the fundamentals behind clinical decisions. Likewise, the clinical implications of midwives’ knowledge scores are likely to be minimal if they are not regularly involved in TB patient care.

The present study was the first to look at knowledge regarding Xpert MTB/RIF®, a relatively new nucleic acid amplification test used for diagnosis of TB and resistance testing, in a low-resource high-TB burden setting. Despite the WHO issuing recommendations endorsing Xpert MTB/RIF® technology in December 2010 and the widespread uptake of its use worldwide since [1], knowledge of its existence and purpose remains low in the present study. The use of Xpert in Mozambique at the time of the survey was limited, although it was available in the district of Manhiça (among others) for specific high risks groups where it has been broadly used as part of surveillance based studies. This perhaps suggests that the roll out of Xpert MTB/RIF® might not have been accompanied with sufficient education for HCWs. While this technology is lauded as “a major advance for TB diagnostics”, without a widespread understanding of its potential among HCWs and the critical operational requirement of trained laboratory and clinical staff, Xpert MTB/RIF® may not be fully adopted [31]. There is a paucity of evidence investigating HCWs’ knowledge regarding paediatric TB. Regardless of demographic characteristics, respondents were not aware of the treatment duration or the diagnostic differences between children and adults. These findings provide impetus for further research into paediatric TB HCWs’ knowledge and education.

The HCWs identified that there was clearly a stigma associated with TB. About a third of HCWs in the present study believed that their clinical conduct did not contribute to this stigma, while, conversely, many believed that their actions did stigmatise TB patients citing their use of personal protective equipment as sufficient to perpetuate the stigma. Two studies from Ghana found that fear of infection is a major cause of TB stigma and this fear results in TB patients being shunned, avoided and segregated [32, 33]. The exact cause of the stigma in the present study warrants further investigation as a review of 83 studies by Chang et al. revealed that there are significant cultural variations with respect to TB and the development of stigma [34]. Attitudes towards traditional and/or alternative medicine were largely negative or neutral. Over 70% of the HCWs disagreed with the statement that “Access to traditional and alternative medicine improves patient wellbeing” and many HCW believed that they were in fact a hindrance to TB management. These sentiments are in line with the conclusions by Islam et al. who showed that allopathic providers lack knowledge and irrationally use antibiotics, adding to the challenge of TB control and prevention [35]. It is unclear whether combining traditional and medical management leads to poorer treatment outcomes [36, 37], as this probably depends on the relationship between formal and informal healthcare providers.

Practice competency as measured by the survey was low. The questions asked were deemed essential knowledge that most HCWs should be familiar with. The low scores may relate to the fact that the survey was administered among all HCWs from the Manhiça district, regardless of profession or department; as such professionals may have a lack of experience in dealing with common TB scenarios. Chakaya et al. assessed practice competency in HCWs with comparable methods, finding similar deficiencies in the initial investigation of TB suspects as well as an unfamiliarity with anti-TB drugs and regimens [27].

This study has several limitations. Firstly, the external validity might be affected by its cross-sectional analysis and relatively small sample size. Seventy per cent of the population in Southern Mozambique lives in rural areas, as such, we believe the Manhiça District might be a reliable reflection of the rest of the region. Secondly, the semi-qualitative analysis approach may have resulted in sections of data being misinterpreted due to thematic aggregation. Thirdly, practice competency was approximated by self-reporting, rather than direct observation, which may overstate adherence to guidelines. Reinforcing the fact that the survey was not an assessment of individual’s knowledge and performance reduced the potential for them to seek external help and affect results. Nonetheless, this study offers novel findings that may be applied to similar settings.

## Conclusion

The assessment of TB HCWs’ knowledge, attitudes and practises provides valuable baseline information concerning insufficiencies and obstacles to sound TB care and control. The present study provides impetus for tailored education among HCWs regarding TB in general, in children and diagnostic testing, such as Xpert MTB/RIF®, as well as reassessment of TB HCW KAP following education. Further qualitative assessment is also indicated to establish the grounding for TB-associated stigma.

## Additional file

Additional file 1: Questionnaire used in the survey (English language). (DOCX 27 kb)

## Abbreviations

ANOVA: Analysis of variance
CISM: Manhiça health research center
CNBS: National bioethics committee
HCC: Health care centers
HCW: Health care workers
HIV: Human immunodeficiency virus
KAP: Knowledge, attitudes, practices
NTP: National tuberculosis program
SD: Standard deviation
TB: Tuberculosis
WHO: World health organization

## References

[1] WHO (2014). Global tuberculosis report.

[2] WHO (2015). Global tuberculosis report.

[3] Brassard P, Anderson KK, Menzies D, Schwartzman K, Macdonald ME (2008). Knowledge and perceptions of tuberculosis among a sample of urban aboriginal people. J Community Health.

[4] Hoa NP, Diwan VK, Thorson AE (2005). Diagnosis and treatment of pulmonary tuberculosis at basic health care facilities in rural Vietnam: a survey of knowledge and reported practices among health staff. Health Policy.

[5] Stop TB Policy Paper: Contributing to Health System Strengthening: Guiding Principles for National Tuberculosis Programmes. Geneva: World Health Organization; 2008 Jan. THE STOP TB STRATEGY. Available from: http://www.who.int/tb/dots/whatisdots/en/index2.html 24921118

[6] Minnery M, Contreras C, Perez R (2013). A cross sectional study of knowledge and attitudes towards tuberculosis amongst front-line tuberculosis personnel in high burden areas of Lima, Peru. PLoS ONE.

[7] Buregyeya E, Kulane A, Colebunders R (2011). Tuberculosis knowledge, attitudes and health-seeking behaviour in rural Uganda. Int J Tuberc Lung Dis.

[8] Haasnoot PJ, Boeting TE, Kuney MO, van Roosmalen J (2010). Knowledge, attitudes, and practice of tuberculosis among maasai in simanjiro district, Tanzania. Am J Trop Med Hyg.

[9] Kanjee Z, Catterick K, Moll AP, Amico KR, Friedland GH, 4 (2011). Tuberculosis infection control in rural south Africa: survey of knowledge, attitude and practice in hospital staff. J Hosp Infect.

[10] Naidoo S, Taylor M, Esterhuizen TM (2011). Changes in healthcare workers’ knowledge about tuberculosis following a tuberculosis training programme. Educ Health (Abingdon).

[11] Roy A, Abubakar I, Chapman A (2011). A controlled trial of the knowledge impact of tuberculosis information leaflets among staff supporting substance misusers: pilot study. PLoS ONE.

[12] Awofeso N, Schelokova I, Dalhatu A (2008). Training of front-line health workers for tuberculosis control: lessons from Nigeria and Kyrgyzstan. Hum Resour Health.

[13] Vanden Driessche K, Sabue M, Dufour W, Behets F, Van Rie A (2009). Training health care workers to promote HIV services for patients with tuberculosis in the democratic republic of Congo. Hum Resour Health.

[14] Pfeiffer J, Chapman R (2015). An anthropology of aid in Africa. Lancet.

[15] Garcia-Basteiro AL, Lopez-Varela E, Manhica I, Macete E, Alonso PL, 9913 (2014). Mozambique faces challenges in the fight against tuberculosis. Lancet.

[16] Garcia-Basteiro AL, Lopez-Varela E, Augusto OJ (2015). Radiological findings in young children investigated for tuberculosis in mozambique. PLoS ONE.

[17] Garcia-Basteiro AL, Lopez-Varela E, Respeito D (2015). High tuberculosis burden among people living with HIV in southern Mozambique. Eur Respir J.

[18] Lopez-Varela E, Augusto OJ, Gondo K (2015). Incidence of tuberculosis among young children in rural Mozambique. Pediatr Infect Dis J.

[19] García-Basteiro AL, Respeito D, Augusto OJ (2016). Poor tuberculosis treatment outcomes in southern Mozambique (2011–2012). BMC Infect Dis.

[20] García-Basteiro AL, Ribeiro RM, Brew J, et al. Tuberculosis on the rise in southern Mozambique (1997–2012). Eur Respir J. 2016; In Publication. 10.1183/13993003.01683-2016 28331035

[21] Sacoor C, Nhacolo A, Nhalungo D (2013). Profile: Manhica Health Research Centre (Manhica HDSS). Int J Epidemiol.

[22] Instituto Nacional de Estatística (2013). Estatísticas Distritais (Estatísticas do Distrito de Manhiça).

[23] Instituto Nacional de Estatística (2010). Projecções anuais da população total, Urbana e rural, dos distritos da província de Maputo 2007 – 2010.

[24] Kanjee Z, Catterick K, Moll AP, Amico KR, Friedland GH (2011). Tuberculosis infection control in rural south Africa: survey of knowledge, attitude and practice in hospital staff. J Hosp Infect.

[25] Heunis C, Wouters E, Kigozi G, Van Rensburg-Bonthuyzen EJ, Jacobs N, 2 (2013). TB/HIV-related training, knowledge and attitudes of community health workers in the free state province, south Africa. Afr J AIDS Res.

[26] Temesgen C, Demissie M (2014). Knowledge and practice of tuberculosis infection control among health professionals in northwest Ethiopia; 2011. BMC Health Serv Res.

[27] Chakaya JM, Meme H, Kwamanga D (2005). Planning for PPM-DOTS implementation in urban slums in Kenya: knowledge, attitude and practices of private health care providers in Kibera slum, Nairobi. Int J Tuberc Lung Dis.

[28] Peltzer K, Mngqundaniso N, Petros G, 6 (2006). HIV/AIDS/STI/TB knowledge, beliefs and practices of traditional healers in KwaZulu-Natal, south Africa. AIDS Care.

[29] Bell CA, Duncan G, Saini B (2011). Knowledge, attitudes and practices of private sector providers of tuberculosis care: a scoping review. Int J Tuberc Lung Dis.

[30] Naidoo S, Taylor M, Esterhuizen TM, 2 (2011). Changes in healthcare workers’ knowledge about tuberculosis following a tuberculosis training programme. Educ Health.

[31] Piatek AS, Van Cleeff M, Alexander H (2013). GeneXpert for TB diagnosis: planned and purposeful implementation. Glob Health Sci Pract.

[32] Dodor EA, Kelly SJ (2010). Manifestations of tuberculosis stigma within the healthcare system: the case of Sekondi-Takoradi metropolitan district in Ghana. Health Policy.

[33] Dodor EA, Neal K, Kelly S (2008). An exploration of the causes of tuberculosis stigma in an urban district in Ghana. Int J Tuberc Lung Dis.

[34] Chang SH, Cataldo JK (2014). A systematic review of global cultural variations in knowledge, attitudes and health responses to tuberculosis stigma. Int J Tuberc Lung Dis.

[35] Islam QS, Ahmed SM, Islam MA, Chowdhury AS, Siddiquea BN, Husain MA (2014). Informal allopathic provider knowledge and practice regarding control and prevention of TB in rural Bangladesh. Int Health.

[36] Rowe KA, Makhubele B, Hargreaves JR, Porter JD, Hausler HP, Pronyk PM (2005). Adherence to TB preventive therapy for HIV-positive patients in rural South Africa: implications for antiretroviral delivery in resource-poor settings?. Int J Tuberc Lung Dis.

[37] Greene JA (2004). An ethnography of nonadherence: culture, poverty, and tuberculosis in urban Bolivia. Cult Med Psychiatry.

