# Supplementary material for: Knowledge, attitudes and practices regarding tuberculosis care among health workers in Southern Mozambique
Source: BMC Pulm Med. 2017 Jan 5;17:2. doi: 10.1186/s12890-016-0344-8 (PMC5217625; doi:10.1186/s12890-016-0344-8)
Supplement: Additional file 1: — Questionnaire used in the survey (English language). (DOCX 27 kb) [file 12890_2016_344_MOESM1_ESM.docx]

# Additional file 1: Questionnaire

Please fill in the blanks and mark the answer that you think best answers the question.

## Demographics

1. What is your age?
   1. __________
2. What is your gender?
   1. Male
   2. Female
3. What is your highest level of educational attainment?
4. Primary School
5. Secondary School
6. Professional Technical training
7. University
8. In which health centre do you currently work? _____________________________
9. What is your profession within said health centre? (for example: doctor, nurse, laboratory staff, activist, medical agent, etc)
10. How long have you worked within said health centre?
    1. Less than one year
    2. One to five years
    3. Five to ten years
    4. More than ten years
11. How long have you cared for patients with tuberculosis or presumptive cases of tuberculosis?
    1. Less than one year
    2. One to five years
    3. Five to ten years
    4. More than ten years
    5. Never
12. How many patients treated for tuberculosis have you cared for in the last 6 monts?
    1. 0 – <10
    2. 10 - <40
    3. >40
13. Are you directly involved in tuberculosis control activities?
    1. Yes
    2. No

If yes, please specify: __________________________________________

1. Have you ever received tuberculosis-specific training?
   1. Yes
   2. No
2. In the past 6 months have you received any tuberculosis-specific training?
   1. Yes
   2. No
3. Have you ever been sick with tuberculosis?
   1. Yes
   2. No
4. Has someone you are in close contact with ever been sick with tuberculosis (for example: family members, spouse, etc)?
   1. Yes
   2. No

## Knowledge

### Tuberculosis Basics

1. What is the causative agent of tuberculosis?
   1. **Mycobacterium tuberculosis**
   2. Mycobacterium avium
   3. Mycobacterium pneumoniae
   4. Mycobacterium leprae
2. Is tuberculosis a transmissible disease?
   1. **Yes**
   2. No
3. How does tuberculosis spread?
   1. Sexually
   2. **By droplet spread**
   3. By direct contact
   4. By sharing needles
4. Which group of people is at higher risk of developing tuberculosis?
   1. Patients with COPD
   2. **Patients with HIV**
   3. Males
   4. People living in tropical areas
5. If a person contracts tuberculosis, what is the likelihood they will develop active tuberculosis within the immediate time frame? (The closest answer will be accepted)
   1. 90%
   2. 60%
   3. 40%
   4. **10%**
6. What type of preventive measures can you take as health care professional if you are dealing with a tuberculosis patient or a suspect of tuberculosis:

Please briefly explain:______________________________________________________

_______________________________________________________________________

1. Have you ever heard of the Tuberculosis Infection Control Plan for your Health Unit?
   1. **Yes**
   2. No
2. If yes, have you received training on its content?
   1. **Yes**
   2. No

### Tuberculosis Diagnosis

1. What is the most common symptom of pulmonary tuberculosis?
2. Haemoptysis
3. Persistent and high fever
4. Loss of weight
5. **Persistent cough**
6. What is the best diagnostic tool for tuberculosis?
7. Blood culture
8. Skin test
9. **Culture**
10. Sputum microscopy
11. TB diagnosis in children is more difficult than in adults?
12. yes
13. no

If yes, briefly explain_________________________________________________________

1. How many sputum samples are necessary for diagnosis?
2. 1
3. **2**
4. 3
5. 4
6. When should the initial sputum sample be taken?
7. After waiting 2 – 3 hours following triage
8. **Immediately**
9. The next morning
10. When the patient has arrived at the laboratory and is ready to give a sample
11. How should a sputum sample be stored before laboratory analysis?
12. In culture medium
13. At room temperature
14. Sputum samples cannot be stored – they should be sent to the laboratory urgently
15. **In the fridge**
16. Do you know what Gene Xpert is?
17. **Yes**
18. No

If yes: What is it? _______________________________________________

### Tuberculosis Treatment

1. Is tuberculosis a curable disease?
2. **Yes**
3. No
4. How long is the first line treatment of pulmonary tuberculosis?
5. 2 months
6. **6 months**
7. 9 months
8. 12 months
9. Does tuberculosis treatment in children have a longer length?
10. **Yes**
11. No
12. How many drugs are used in the first line treatment of tuberculosis?
13. 1
14. 2
15. 3
16. **4**
17. Do you know what direct observed treatment during the initial phase of treatment is?
18. **Yes**
19. No

If yes, explain briefly:__________________________________________

___________________________________________________________

1. What is multi-drug resistant tuberculosis?
2. **Tuberculosis that is resistant to Isoniazid and Rifampicin**
3. Tuberculosis that is resistant to any one tuberculosis medication
4. Tuberculosis that is resistant to all tuberculosis medications
5. Tuberculosis that is resistant to Pyrazinamide and Ethambutol
6. In which group of people is multi-drug resistant tuberculosis most likely to occur?
7. In people with HIV
8. In people who have been exposed to other tuberculosis patients
9. **In people who have been treated for tuberculosis before**
10. In people who have never had tuberculosis before
11. When should the first follow up sputum sample be carried out following the commencement of treatment of a confirmed case of tuberculosis?
12. 1 month after the commencement of treatment
13. **2 month after the commencement of treatment**
14. 3 month after the commencement of treatment
15. 7 month after the commencement of treatment
16. What is the major element to assess tuberculosis treatment cure?
17. Skin test
18. Chest X-ray
19. **Sputum**
20. All of the above
21. What are the consequences of incomplete treatment?
22. Development of resistant tuberculosis
23. Failure to fully cure the disease
24. Further transmission of the disease
25. **All of the above**
26. Is there a vaccine for TB?
27. **Yes**
28. No

If yes, name it:__________________________________________

___________________________________________________________

1. When is the best time for BCG vaccination?
2. **At birth**
3. At 2 months
4. At 6 months
5. Other. Specify __________________________________

## Attitudes

Please mark which most applies regarding your attitude to the statement. If the statement does not apply to you do not tick a box.

1. Finding every new case of tuberculosis is essential for control of the disease
   1. Strongly agree
   2. Agree
   3. Neutral
   4. Disagree
   5. Strongly Disagree
2. Community engagement is essential for the control of the disease
   1. Strongly agree
   2. Agree
   3. Neutral
   4. Disagree
   5. Strongly Disagree
3. There is a substantial increase in treatment completion rates if direct observed treatment is used
   1. Strongly agree
   2. Agree
   3. Neutral
   4. Disagree
   5. Strongly Disagree
4. There is a stigma associated with tuberculosis in Mozambique
   1. Strongly agree
   2. Agree
   3. Neutral
   4. Disagree
   5. Strongly Disagree

Briefly explain: ____________________________________________________________

____________________________________________________________

1. The way you interact with tuberculosis patients/regard tuberculosis contributes any possible stigma there is
   1. Strongly agree
   2. Agree
   3. Neutral
   4. Disagree
   5. Strongly Disagree

Briefly explain: _____________________________________________________________

_____________________________________________________________

1. Tuberculosis as a disease has more stigma associated with it than HIV
   1. Strongly agree
   2. Agree
   3. Neutral
   4. Disagree
   5. Strongly Disagree
2. Money spent on educating the Mozambican general population is better than money spent on direct observed treatment
   1. Strongly agree
   2. Agree
   3. Neutral
   4. Disagree
   5. Strongly Disagree
3. In Mozambique, the general population is aware of the tuberculosis services that are available
   1. Strongly agree
   2. Agree
   3. Neutral
   4. Disagree
   5. Strongly Disagree
4. Public awareness regarding tuberculosis as a health problem in Mozambique is adequate
   1. Strongly agree
   2. Agree
   3. Neutral
   4. Disagree
   5. Strongly Disagree

Briefly explain: ______________________________________

____________________________________________________________

1. Multi-drug resistant tuberculosis is a problem in Mozambique
   1. Strongly agree
   2. Agree
   3. Neutral
   4. Disagree
   5. Strongly Disagree
2. Traditional or alternative medicine assists in wellbeing of tuberculosis patients
   1. Strongly agree
   2. Agree
   3. Neutral
   4. Disagree
   5. Strongly Disagree
3. First line therapies for tuberculosis are accepted by patients
   1. Strongly agree
   2. Agree
   3. Neutral
   4. Disagree
   5. Strongly Disagree
4. In Mozambique, there are many barriers to tuberculosis treatment
   1. Strongly agree
   2. Agree
   3. Neutral
   4. Disagree
   5. Strongly Disagree

Briefly explain: _______________________________________________

____________________________________________________________

1. The majority of staff in your health centre have adequate training regarding tuberculosis
   1. Strongly agree
   2. Agree
   3. Neutral
   4. Disagree
   5. Strongly Disagree
2. The laboratory service that your health centre uses is adequate for the diagnosis of tuberculosis
   1. Strongly agree
   2. Agree
   3. Neutral
   4. Disagree
   5. Strongly Disagree
3. In your health centre, there is a sufficient number of people required to treat the tuberculosis patients seen
   1. Strongly agree
   2. Agree
   3. Neutral
   4. Disagree
   5. Strongly Disagree
4. Making people with suspected/confirmed pulmonary tuberculosis wear masks in the hospital is acceptable
   1. Strongly agree
   2. Agree
   3. Neutral
   4. Disagree
   5. Strongly Disagree
5. Teaching tuberculosis patients cough hygiene is not important
   1. Strongly agree
   2. Agree
   3. Neutral
   4. Disagree
   5. Strongly Disagree
6. Infection control is an important means to prevent contracting tuberculosis
   1. Strongly agree
   2. Agree
   3. Neutral
   4. Disagree
   5. Strongly Disagree
7. I have been seriously concerned I have had tuberculosis
   1. Strongly agree
   2. Agree
   3. Neutral
   4. Disagree
   5. Strongly Disagree
8. I should know whether I’ve got or had tuberculosis
   1. Strongly agree
   2. Agree
   3. Neutral
   4. Disagree
   5. Strongly Disagree
9. If I contracted tuberculosis, I would be allowed to continue working in my current capacity
   1. Strongly agree
   2. Agree
   3. Neutral
   4. Disagree
   5. Strongly Disagree
10. My employer would maintain confidentiality if I were to contract tuberculosis
    1. Strongly agree
    2. Agree
    3. Neutral
    4. Disagree
    5. Strongly Disagree
11. I should know my own HIV status
    1. Strongly agree
    2. Agree
    3. Neutral
    4. Disagree
    5. Strongly Disagree

Briefly explain: _______________________________________________

____________________________________________________________

## Practices

1. A patient comes in with a persistent cough of 16 days duration, the cough is dry and the patient is not short of breath. The patient is experiencing night sweats, fatigue and lives with his brother that has been feeling the same way for the past four weeks.
   1. What is the most likely differential diagnosis and what one diagnostic test would you do to exclude your most likely differential diagnosis?
      1. **Tuberculosis**
      2. **Sputum sample, immediately**
   2. What drug(s) would you use and for how long would you use them in the initial phase of tuberculosis treatment?
      1. **Pyrazinamide, Isoniazid, Rifampicin and Ethambutol**
      2. **2 months**
   3. What drug(s) would you use and for how long would you use them in the continuation phase of tuberculosis treatment?
      1. **Isoniazid and Rifampicin**
      2. **4 months**
2. What would you do if you have a patient that grows a positive sputum sample following four months of treatment?
   1. **Start new first line treatment**
   2. Continue since last intake
   3. I do not know
3. What should you do if your patient has yellowish skin after three weeks of treatment?
   1. Consider this as a potential side effect of drug therapy
   2. Stop treatment
   3. Evaluate level of liver enzymes if possible
   4. **All of the above are correct**
